# Artificial Intelligence in medical imaging practice: looking to the future

**DOI:** 10.1002/jmrs.369

**Published:** 2019-11-10

**Authors:** Sarah J Lewis, Ziba Gandomkar, Patrick C Brennan

**Affiliations:** ^1^ Discipline of Medical Imaging Science The University of Sydney Lidcombe New South Wales Australia

## Abstract

Artificial intelligence (AI) is heralded as the most disruptive technology to health services in the 21^st^ century. Many commentary articles published in the general public and health domains recognise that medical imaging is at the forefront of these changes due to our large digital data footprint. Radiomics is transforming medical images into mineable high‐dimensional data to optimise clinical decision‐making; however, some would argue that AI could infiltrate workplaces with very few ethical checks and balances. In this commentary article, we describe how AI is beginning to change medical imaging services and the innovations that are on the horizon. We explore how AI and its various forms, including machine learning, will challenge the way medical imaging is delivered from workflow, image acquisition, image registration to interpretation. Diagnostic radiographers will need to learn to work alongside our ‘virtual colleagues’, and we argue that there are vital changes to entry and advanced curricula together with national professional capabilities to ensure machine‐learning tools are used in the safest and most effective manner for our patients.

## Graphical abstract

This article summarises the opportunities for artificial intelligence (AI) translation into medical imaging in the near future. The changing roles for diagnostic radiographers are explored, and a discussion of the challenges for the ethical implementation of AI is included.

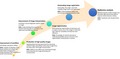

For many of us, our perception of artificial intelligence (AI) is shaped by our exposure to film and television media. For the baby boomers, this may be the stilted, sarcastic robot from Lost in Space and for Generation Xs’, the galactic lifestyles of The Jetsons told us what the future would look like. Millennials have been exposed to the mega‐mane, The Terminator and already live in a social media world influenced by algorithms. It seems for a long time we have known that the intelligence of artificial (non‐human) computer‐based technologies was coming our way. Together with the explosion of AI‐related professional discourse in medical imaging over the last 5 years, in 2019, Price Waterhouse Coopers have heralded unprecedented spending on AI technologies over the next 10 years.^1^ Machine learning (ML) has arrived into our working world, and medical radiation practitioners have the front seat on this bandwagon.

There is widespread acknowledgement that AI will transform the healthcare sector, particularly diagnosis in the field of medical imaging.^2^, ^3^ Further extension into AI‐driven advances in health prevention, precision and management is on the horizon by combining radiomics from medical images with other data forms such as genomics, proteomics and demographics. Within medical imaging, we are seeing implementation of AI tools introduced at a local level to reduce labour intensive and repetitive tasks such as analysis of medical images.^2^ As our information systems grow in their capacity to harvest big data, so has the scope to build AIs in areas such as natural language processing (NLP).

Machine learning (ML) refers to a system that has the capacity to ‘improve’ and ‘learn’ to recognise patterns of disease features, such as the appearance of breast cancers on mammograms as well as analysing surrounding textural features.^4^ Many AI‐aided diagnostic tools for cancer detection applied in recent years have demonstrated excellent progress, especially in areas such as screening mammography, lung cancer screening and histopathological breast images. Studies on the implementation of AI for lung pathology^5^, ^6^ and breast cancer detection^7^ have documented comparable sensitivity and specificity scores for AI tools, either as stand‐alone readers or when combined with radiologists’ scores. In Australia, researchers from the University of Sydney have developed and tested the ability of AI in categorising breast histopathological images as benign or malignant achieving an accuracy of 98.77%.^8^ Indeed for medical imaging, the diagnostic challenge is to move beyond screening test sets and apply the power of algorithms to take into account variations in patient characteristics, disease incidence and severity. Investment is currently underway in developing personalised cancer risk assessment and screening for breast cancer through deep learning models, with the authors of this paper currently receiving funding from the National Health and Medical Research Council (NHMRC) for such an AI project.

Although image interpretation is possibly the most well‐researched task in medical imaging where AI has been applied^4^, ^5^, ^6^, ^7^, AIs have recently been adopted in other areas of practice such as medical image denoising, dose reduction, autosegmentation, case triage and image reconstruction. The span of AI pathways in medical imaging is shown in Figure 1. An example of this practice is demonstrated in a study by Wolterink et al., where AI was used to estimate routine‐dose computed tomography (CT) images from low‐dose CT images^9^ while Wang et al.^10^ proposed an AI‐based tool to estimate the high‐quality full‐dose positron emission tomography (PET) images from low‐dose images. Utilising these AI tools could ultimately lead to a reduction in the radiation exposure while maintaining the high quality of medical images, although risks such as image distortion must be assessed.

**Figure 1:**
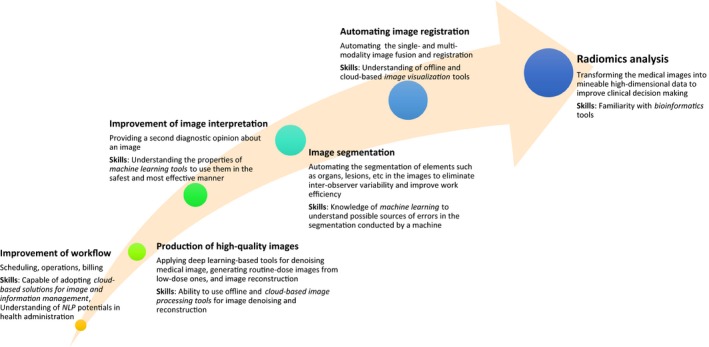
AI areas of impact for medical imaging practice.

A possible role for diagnostic radiographers could be at the forefront of developing and validating low‐dose CT protocols that can be ‘converted’ to standard dose imaging by increasing their skills in visual image analysis. Unlike analytical or iterative image reconstruction techniques which require expert knowledge to optimise reconstruction performance, deep learning‐based methods reforms image reconstruction as a data‐driven supervised learning problem of finding a mapping between the sensor and the image domain. As an example, Zhu et al.^11^ showed that a deep learning‐based method is more robust to noise and exhibited a significant reduction in reconstruction artefacts compared with conventional reconstruction methods. In another study, Kim et al.^12^ utilised deep learning to construct high‐resolution magnetic resonance (MR) images in one contrast from highly down‐sampled MR images in another contrast.

Deep learning has huge potential in optimising image registration, which is essential in many clinical tasks such as investigating longitudinal changes or image fusion. As an example, Cheng et al. recently proposed a deep learning‐based model for coregistering the CT and magnetic resonance (MR) images.^13^ In another study, Wu et al. showed that a deep learning‐based method for registering the brain MR images outperformed all state‐of‐the‐art deformable image registration methods.^14^ AI has also been used to fast track reporting of CT cases acquired for aortic injury by using a deep learning Convolutional Neural Network (CNN). In this research, the CNN was verified by the NLP of radiology reports to determine the effectiveness of using AI for study prioritisation into radiologists’ workflow.^15^

Automatic segmentation is a key AI innovation in oncology imaging with improved accuracy of contouring for organs at risk (OAR) in radiotherapy planning, removing interobserver variability and improving work efficiency.^16^ As an example, Tang et al. proposed a deep learning‐based tool to semi‐automatically provide the response evaluation criteria in solid tumours (RECIST), which is currently used as a standard measurement for tumour extent to assess patients’ treatment.^17^ The system was shown to be unaffected by inter‐reader variability while demonstrating a promising agreement with radiologists’ consensus. In this issue of the Journal of Medical Radiation Sciences, we read about the national implementation of atlas‐based auto segmentation for radiation therapy (ABAS), whereby morphologic atlases from a data bank of previously contoured scans are applied by an AI tool to optimally segment organs. The article by Hu et al.^18^ shares with readers the benefits and limitations of introducing AI into the radiation therapy workflow, showing that Australian departments are early adopters of such technology with local workplace radiation therapists acting as ‘champions’.

For medical imaging practitioners, the future that includes an ‘AI work colleague’ may represent a scary or exciting concept. Diagnostic radiographers will have an important role to play in building quality imaging biobanks, the databases that feed the AIs and the development of national systems that collect and manage these repositories. In the same way that AI is being developed to provide personalised quantification of risk of disease or wellness, AIs can be developed to personalise imaging protocols for modalities such as CT, MRI and molecular imaging and this is where diagnostic radiographers may adopt a role in optimising AIs.^19^ A further important capability is the ability to recognise the limitations and biases of AIs and to identify and apply its best features in an ethically appropriate way. AIs, like all trained machine learning, have the capability to lead to adverse outcomes for patients and the European Society of Radiology states the ‘challenge for humans is to anticipate how AI systems can go wrong or could be abused and to build in protections against this happening’.^19^

A current hot topic for many healthcare forums today is *will a robot take my job?* It is both an amusing yet serious consideration for medical imaging professionals. There is no obvious answer to such a question except the need to be on the front foot to prepare for our changing roles. Indeed, Pearce et al. in the Medical Journal of Australia note that ‘decision support tools driven by artificial intelligence are a new clinical method that clinicians need to embrace’ rather than fear and identifies that radiology is one field where human replacement may be under threat from AI.^20^ In order to ensure tomorrow's medical imaging and radiation therapy leaders, and today's current students at all levels: undergraduate, postgraduate and advanced practice, are ready, it is imperative that AI knowledge is integrated into the medical radiation practice curriculum. Sounds too far into the future? Remember that 20 years ago, a PACS radiographer was a novel inclusion to the workforce.

Figure 1 identifies many inclusions or improvements to current curricula that the modern medical imaging professional will need to participate in a workplace that includes AI. These include greater education that promotes understanding of ML and NLP to manage image and information management. Diagnostic radiographers in the future also need to have the skills to use offline and cloud‐based tools for image processing, visualisation, reconstruction and in addition to ability to recognise potential errors produced by ML through the incorrect application of algorithms, such as in image segmentation.

In the era of personalised and precision medicine, there is a growing interest in transforming medical images into mineable high‐dimensional data or radiomic features which can be used to improve clinical decision‐making.^21^ Usually radiomics are fed into bioinformatic tools to explore their diagnostic, prognostic and predictive potentials. As analysing radiomic features are ultimately intended to be undertaken in routine clinical practice,^21^ radiographers should become familiar with the process and tools used for the conversion of digital images to mineable data and issues that may occur due to interscanner and intervendor variability. Radiographers who are equipped with bioinformatic skills can be leaders in harnessing the power of radiomics for facilitating improved clinical decision‐making.

Medical Radiation Science (MRS) academics must be fluent in the disruptive technologies of AI so they can prepare students for a workforce where AI will be their colleague, and where traditional models of decision‐making will be altered to include the integration of machine learning. All of this comes with a requirement for greater capability in computing science as well as image analysis and processing. Future revision of the Professional Capabilities for Medical Radiation Practitioners^22^ will need to acknowledge the need for these new skills and future Codes of Conduct should recognise the role medical imaging practitioners will play in the ethical application of AI for health care.

Finally, it is important to state that emotional intelligence, not artificial intelligence, must always be at the heart of patient care and be central to strategies that move our profession forward. No one wants their radiograph taken by The Terminator.
